# Case Report: A rare case of multicentric angiosarcomas of bone mimicking multiple myeloma on ^18^F-FDG PET/CT

**DOI:** 10.3389/fmed.2023.1330341

**Published:** 2023-11-28

**Authors:** Wenpeng Huang, Xiaoyan Xiao, Yongbai Zhang, Yushuo Peng, Lele Song, Liming Li, Jianbo Gao, Lei Kang

**Affiliations:** ^1^Department of Nuclear Medicine, Peking University First Hospital, Beijing, China; ^2^Department of Radiology, The First Affiliated Hospital of Zhengzhou University, Zhengzhou, Henan Province, China

**Keywords:** bone, angiosarcoma, magnetic resonance imaging, computed tomography, ^18^F-FDG PET/CT, case report

## Abstract

**Background:**

Angiosarcoma, a rare endothelial-origin tumor, can develop throughout the body, with the head and neck skin being the most commonly affected areas. It can also originate in other sites such as the breast, iliac artery, and visceral organs including the liver, spleen, and kidneys. Angiosarcoma of the bone is remarkably rare, presenting as either unifocal or multifocal bone lesions and often leading to a grim prognosis. Diagnosing bone angiosarcoma poses a significant challenge. ^18^F-FDG PET/CT serves as a reliable and indispensable imaging modality for evaluating distant metastases and clinically staging angiosarcomas.

**Case report:**

A 57-year-old woman presented with a 10-day history of dizziness and headaches. Cranial CT scan revealed bone destruction of the parietal bone, accompanied by soft tissue lesions, protruding into the epidural space. MRI examination demonstrated lesions with slightly elevated signal intensity on T2FLAIR, showing moderate enhancement. Furthermore, multiple foci were observed within the T_12_, L_1-5_, and S_1-2_ vertebrae, as well as in the bilateral iliac bones. For staging, ^18^F-FDG PET/CT was performed. The MIP PET showed multifocal FDG-avid lesions in the sternum, bilateral clavicles, bilateral scapulae, multiple ribs, and pelvic bones. Heterogeneous FDG uptake was observed in multiple bone lesions, including intracranial (SUVmax = 11.3), right transverse process of the T10 vertebra (SUVmax = 5.8), ilium (SUVmax = 3.3), and pubis (SUVmax = 4.7). The patient underwent surgical resection of the cranial lesion. The pathological diagnosis was made with a highly differentiated angiosarcoma.

**Conclusion:**

Angiosarcoma of bone on FDG PET/CT scans is characterized by abnormal FDG uptake along with osteolytic destruction. This case highlights that angiosarcoma of bone can manifest as multicentric FDG uptake, resembling the pattern seen in multiple myeloma. FDG PET/CT can be a useful tool for staging this rare malignant tumor, offering the potential to guide biopsy procedures toward the most metabolically active site. And it should be considered in the differential diagnosis of multiple osteolytic lesions, including metastatic carcinoma, multiple myeloma, and lymphoma of bone.

## Introduction

Angiosarcoma is a rare endothelial-origin tumor that can develop in the whole body (1). The most commonly affected areas are the head and neck skin. Other sites where may originate include the breast, iliac artery, and visceral organs such as the liver, spleen and kidneys (2–7). Angiosarcoma of the bone is remarkably rare, constituting less than 1% of all primary bone sarcomas, predominantly occurring between the ages of 50 and 70 (8, 9). It can manifest as either unifocal or multifocal bone lesions and typically heralds a bleak prognosis (10, 11). Diagnosing angiosarcoma of the bone poses a considerable challenge and represents the malignant end of the CD31/ERG positive vascular tumors spectrum, encompassing hemangiomas, hemangioendotheliomas, as well as well-differentiated and poorly differentiated angiosarcomas (12).

Fluorine-18 fluorodeoxyglucose positron emission tomography/computed tomography (^18^F-FDG PET/CT) stands as a dependable and indispensable imaging modality for assessing distant metastases and clinically staging angiosarcomas (13). In an effort to enrich comprehension of this rare neoplasm, we present a case study delineating the ^18^F-FDG PET/CT imaging manifestations in a patient afflicted by multicentric angiosarcomas of the bone, marked by a challenging clinical diagnosis.

## Case presentation

**Figure 1:**
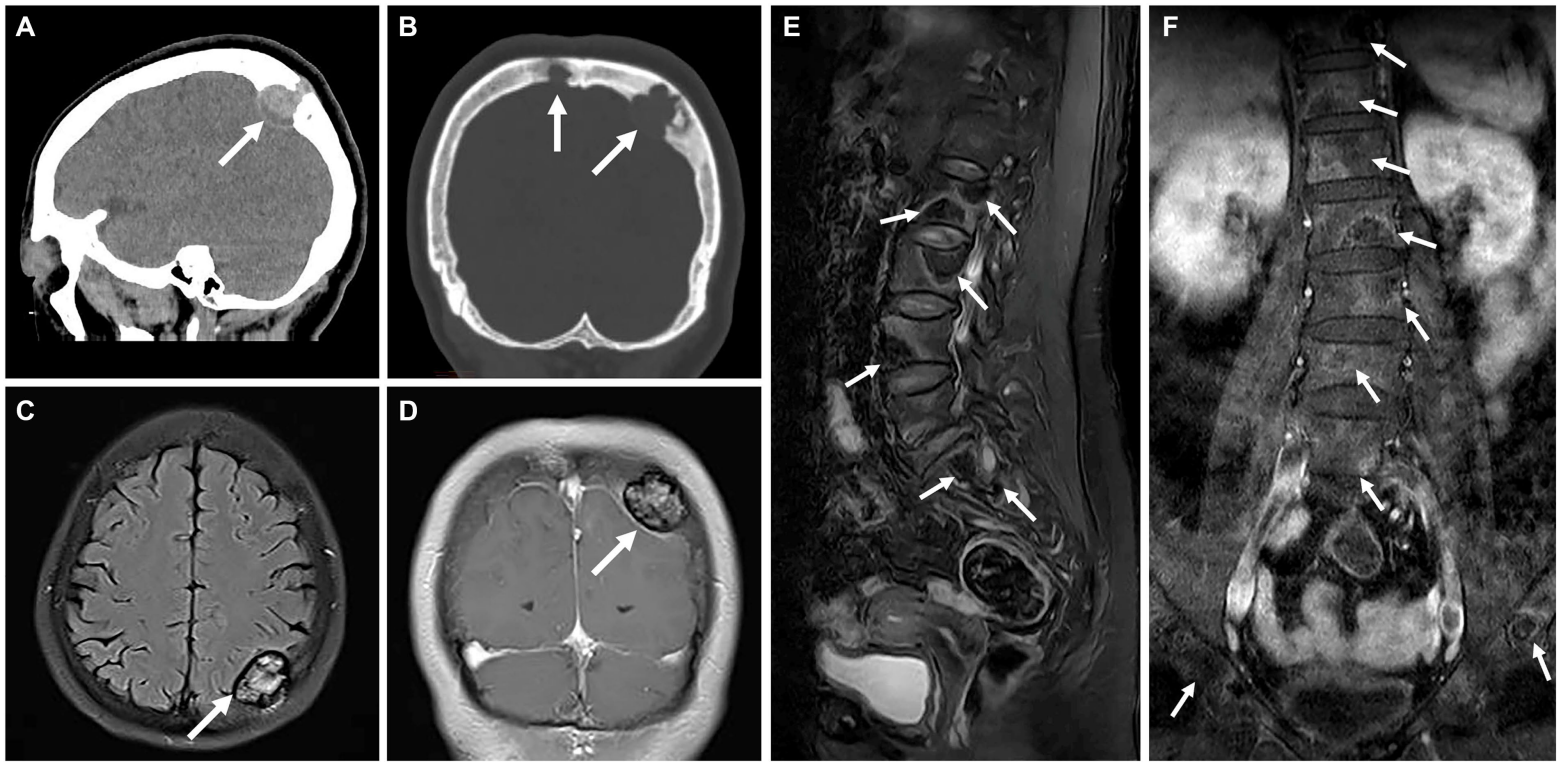
Computed tomography (CT) and magnetic resonance (MR) images of multicentric angiosarcomas of bone. Sagittal and coronal CT image revealed bone destruction of the parietal bone **(A)**, accompanied by soft tissue lesions (long arrows), with the largest measuring approximately 3.6 × 2.7 × 2.0 cm, protruding into the epidural space **(B)**. MRI examination demonstrated lesions with slightly elevated signal intensity on T2FLAIR **(C,** long arrows**)**, showing moderate enhancement on Fat-sat Gd-T1WI **(D)**. Multiple foci were observed within the T12, L1-5, and S1-2 vertebrae, as well as in the bilateral iliac bones. These exhibited irregular signal patterns on fat-suppressed T2WI **(E,** long arrows**)** and mild enhancement on Fat-sat Gd-T1WI **(F)**.

A 57-year-old woman presented with a 10-day history of dizziness and headaches. Cranial CT scan revealed bone destruction of the parietal bone, accompanied by soft tissue lesions, with the largest measuring approximately 3.6 × 2.7 × 2.0 cm, protruding into the epidural space (Figures 1A,B). MRI examination demonstrated lesions with slightly elevated signal intensity on T2FLAIR (Figure 1C), showing moderate enhancement (Figure 1D). Furthermore, multiple foci were observed within the T_12_, L_1-5_, and S_1-2_ vertebrae, as well as in the bilateral iliac bones. These exhibited irregular signal patterns on fat-suppressed T2WI and mild enhancement (Figures 1E,F).

**Figure 2:**
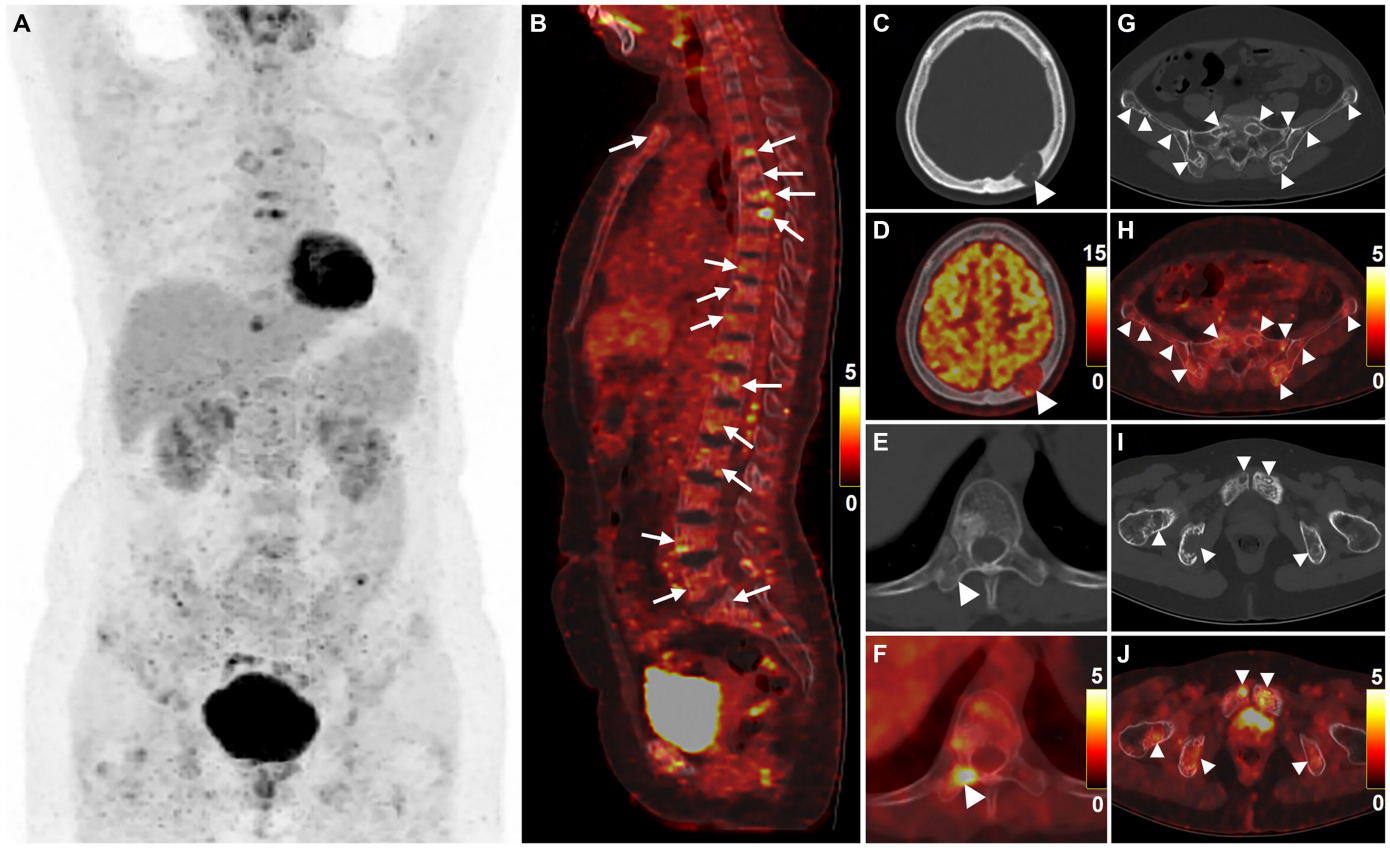
^18^F-FDG PET/CT images of multicentric angiosarcomas of bone. The anteroposterior 3-dimensional maximum intensity projection image (MIP) revealed multifocal FDG-avid lesions in the sternum, bilateral clavicles, bilateral scapulae, multiple ribs, and pelvic bones **(A)**. Sagittal images of the spine revealed the presence of multiple bone-destroying lesions (long arrows), with the highest FDG uptake detected in the T6 vertebra (**B**, SUVmax = 7.6). Heterogeneous FDG uptake was observed in multiple bone lesions (arrowheads), including intracranial (**C,D**, SUVmax = 11.3), right transverse process of the T10 vertebra (**E,F**, SUVmax = 5.8), ilium (**G,H**, SUVmax = 3.3), and pubis (**I,J**, SUVmax = 4.7).

For staging, ^18^F-FDG PET/CT was performed (Figures 2A–J). The MIP PET showed multifocal FDG-avid lesions in the sternum, bilateral clavicles, bilateral scapulae, multiple ribs, and pelvic bones. Sagittal images of the spine revealed the presence of multiple bone-destroying lesions, with the highest FDG uptake detected in the T6 vertebra (SUVmax = 7.6). Heterogeneous FDG uptake was observed in multiple bone lesions, including intracranial (SUVmax = 11.3), right transverse process of the T10 vertebra (SUVmax = 5.8), ilium (SUVmax = 3.3), and pubis (SUVmax = 4.7).

**Figure 3:**
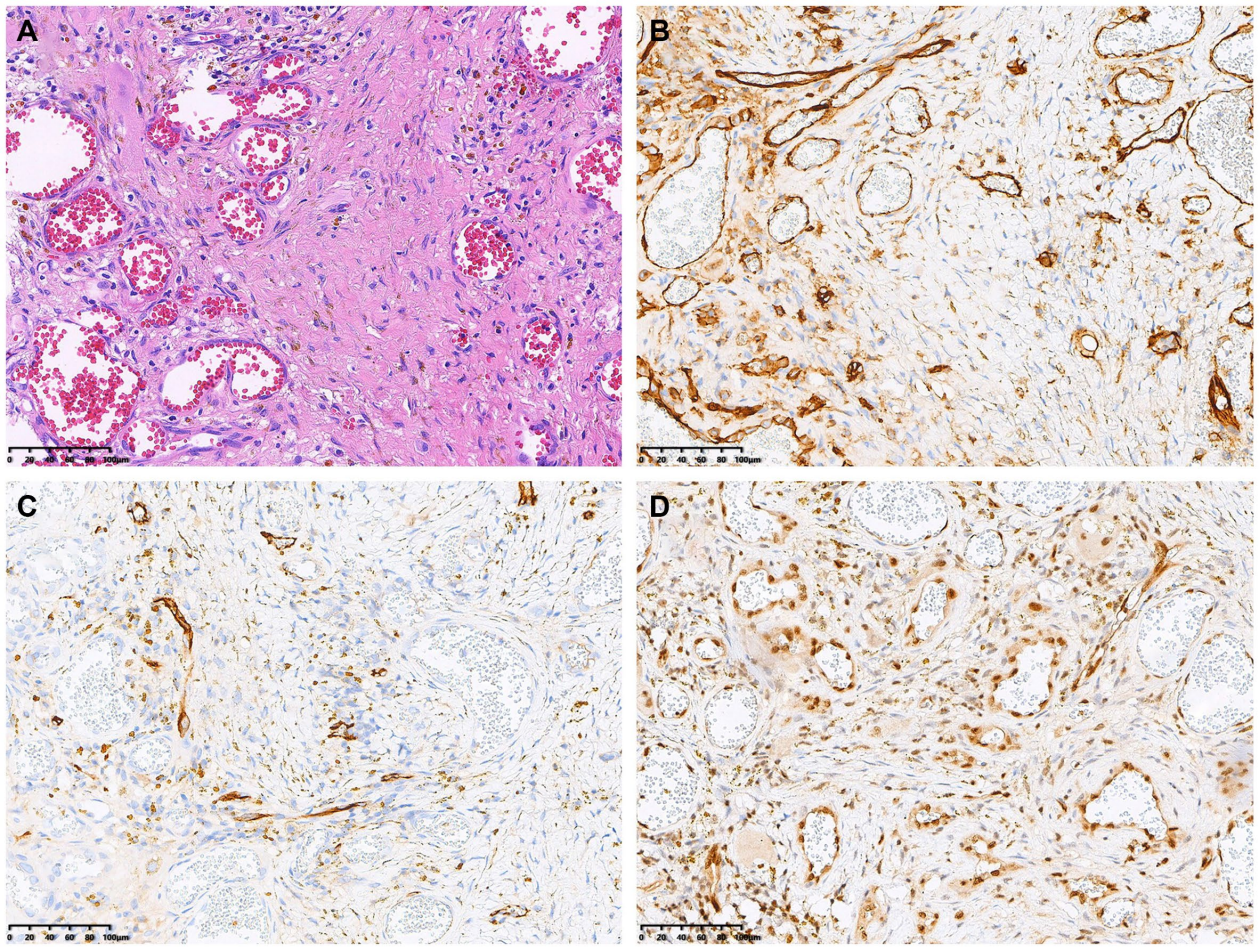
Histopathological and immunohistochemical images. Hematoxylin–eosin (HE) staining (magnification × 200) revealed irregular vascular channels and hyperplasia of atypical cells arranged in a diffuse sheet-like growth pattern **(A)**. Immunohistochemistry showed that the positive expression of CD31 **(B)**, CD34 **(C)** and FLI-1 **(D)** of the tumor cells (magnification × 200).

The patient underwent a bone marrow aspiration biopsy, which showed active proliferation of bone marrow tissue and the presence of granulocyte lineage, thereby ruling out the diagnosis of multiple myeloma. Subsequently, the patient underwent surgical resection of the cranial lesion. Hematoxylin and eosin staining revealed irregular vascular channels and hyperplasia of atypical cells arranged in a diffuse sheet-like growth pattern (Figure 3A). Immunohistochemical staining showed positive expression of CD31 (Figure 3B), CD34 (Figure 3C), ERG, FLI-1 (Figure 3D), and SMA. The pathological diagnosis was made with a highly differentiated angiosarcoma.

## Discussion

Angiosarcomas, highly aggressive and exceptionally rare within the spectrum of sarcomas, represent less than 1% of all sarcomas (4). These sarcomas predominantly afflict elderly men, with over 50% of cases manifesting in the cutaneous and soft tissues of the head and neck. The remaining occurrences of angiosarcomas may emerge from various sites, including the breast, soft tissues, bones, and visceral organs such as the liver and spleen (1, 4, 14, 15). Long-term sun exposure, radiation exposure, genetic predispositions, environmental factors, and trauma have been implicated in the development of angiosarcoma (16).

Vascular bone tumors encompass a spectrum of clinicopathological entities, ranging from benign hemangiomas at one end to angiosarcomas at the other (8, 17). Compared to hemangiomas, recognized as benign tumors, epithelioid hemangioendothelioma represents a low-grade malignancy, and hemangioendothelioma occupies the intermediate category, while angiosarcoma emerges as a high-grade malignancy (18). Angiosarcoma of bone may present as unifocal or multifocal disease, potentially affecting any bone. In the majority of cases, angiosarcoma of bone occurs in long bones and short tubular bones, most frequently observed in the femur, tibia, and humerus, followed by the pelvis, ribs, and vertebrae (8, 17). It can consist of multiple lesions within a single bone, affecting the same extremity, or spreading throughout the skeleton (8, 10, 11, 19). Clinical manifestations typically involve bone pain, pathologic fractures, and hypercalcemia. In our case, the sternum, bilateral clavicles, bilateral scapulae, multiple ribs, and pelvic bones demonstrated multicentricity of the tumor.

The diagnosis of angiosarcoma heavily relies on biopsy findings, with neoplastic cells demonstrating endothelial differentiation of vascular or lymphatic origin. Angiosarcomas exhibit varying histologic presentations, ranging from well-differentiated to poorly differentiated tumors. Histologic characteristics span from abnormal endothelial cells retaining some degree of well-differentiated vascular architecture to poorly differentiated sheets of abnormal cells with substantial hemorrhage and necrosis (16, 20). The most prevalent histological feature observed was the presence of intracytoplasmic vacuoles, either containing erythrocytes or empty. Additionally, the identification of three or more mitoses per 10 high-power fields (HPF), the presence of a macronucleolus, and fewer than five eosinophilic granulocytes per 10 HPF serve as prognostic risk factors. The presence of all three risk factors in a single lesion decreases the 5-year survival rate to 0% (8). Immunohistochemistry plays a vital role in identifying poorly differentiated tumors. Positive staining for the ERG endothelial marker, factor VIII, CD31, FLI-1, CD99, S-100 protein, STAT6, SMA, and the Ki-67 proliferation marker is characteristic for angiosarcomas. Expression of these markers confirms the endothelial phenotype of malignant vascular tumors. Definitive identification relies on immunohistochemistry demonstrating vascular and endothelial markers, such as CD31, CD34, FLI-1, and ERG. Our patient’s tumor displayed positivity for all these markers, with CD31 often considered the most specific marker for vascular bone tumors (8, 21, 22).

The integrated ^18^F-FDG PET/CT stands as the foremost functional and metabolic imaging technique, delivering clinicians with comprehensive and precise information at present (23, 24). On FDG PET scans, angiosarcoma typically exhibits increased FDG uptake, and PET/CT is a valuable tool for staging angiosarcoma and assessing distant metastases (5–7, 10, 25–29). Chen et al. (30) conducted a retrospective study involving 19 pathologically diagnosed angiosarcoma cases before treatment, examining the relationship among clinical characteristics, laboratory examinations, ^18^F-FDG PET/CT parameters, and the prognosis of angiosarcoma. In comparison to conventional imaging, systemic ^18^F-FDG PET/CT, with its high sensitivity and specificity, presents significant advantages in the evaluation of angiosarcoma, particularly in the detection of occult metastases like those in bone marrow, subcutaneous tissue, liver, and even hydrothorax and ascitic fluid. The SUVmax of angiosarcoma correlates with histopathological tumor grade (27). An increasing SUVmax is associated with a worse prognosis, can be a valuable noninvasive prognostic marker (6). Lee et al. (28) observed that the degree of FDG uptake, as reflected by SUVmax, served as a prognostic indicator for patients with vascular tumors. Using a cutoff SUVmax of 3.0, the 2-year progression-free survival rate was notably higher in the 14 patients with a tumor SUVmax <3.0 (75.0%) compared to the 12 patients with a tumor SUVmax ≥3.0 (0%) (*p* = 0.0053). In a study of 16 angiosarcoma cases by Kato et al. (27), higher SUVmax, MTV, whole-body TLG, TBR, and whole-body TLG ratio significantly correlated with poorer overall survival in patients evaluated by ^18^F-FDG PET/CT before treatment. Umemura et al. (29) conducted an early prognosis assessment of 18 cases of cutaneous angiosarcoma using PET/CT, noting that those with higher SUVmax at the initial diagnosis experienced a markedly poorer prognosis compared to those with a lower SUVmax.

The prognosis of angiosarcomas is notably poor (19), with frequent occurrences of both local recurrence and distant metastasis (31, 32), typically resulting in a reported 5-year survival rate of approximately 31–33% (8, 33). Angiosarcoma manifesting in bone exhibits a significantly bleaker prognosis compared to general angiosarcoma, with a 5-year survival rate of 20% and an approximate median survival time of 10 months (34, 35). Wang et al. (11) observed that the prognosis of malignant bone angiosarcoma was notably worse than that of malignant vascular tumors, with a median overall survival (OS) of merely 9 months. Age, stage, and the utilization of surgery stand as independent prognostic factors for patients with bone angiosarcoma (11, 36, 37). Managing angiosarcoma poses a considerable clinical challenge due to both the aggressive nature of the tumor and the limited evidence guiding treatment modalities and agents. Treatment strategies for angiosarcomas are contingent on the stage and location. Surgical resection remains the primary therapeutic approach for localized disease, although achieving negative margins can be challenging due to the infiltrative nature of the ailment. A multidisciplinary approach, involving surgery, radiation, chemotherapy, and potentially recent immune-oncology agents, can yield positive outcomes (38). Studies have suggested that bone angiosarcoma treated with surgery alone or in combination with radiotherapy exhibits better outcomes compared to cases treated with radiotherapy alone or without therapy. Due to the endothelial origin of angiosarcomas, there is a growing interest in employing antiangiogenic agents for this tumor. Research has explored various agents, including bevacizumab, a monoclonal antibody targeting vascular endothelial growth factor-A; TRC105, a monoclonal antibody against endoglin; trebananib, a neutralizing peptibody to angiopoietin-1/2; and vascular endothelial growth factor receptor inhibitors such as pazopanib, sorafenib, and axitinib (16).

## Conclusion

In conclusion, we offer a case study detailing the ^18^F-FDG PET/CT imaging manifestations in a patient affected by multicentric angiosarcomas of the bone. Angiosarcoma of bone on FDG PET/CT scans is characterized by abnormal FDG uptake along with osteolytic destruction. This case highlights that angiosarcoma of bone can manifest as multicentric FDG uptake, resembling the pattern seen in multiple myeloma. FDG PET/CT can be a useful tool for staging this rare malignant tumor, offering the potential to guide biopsy procedures toward the most metabolically active site. And it should be considered in the differential diagnosis of multiple osteolytic lesions, including metastatic carcinoma, multiple myeloma, and lymphoma of bone.

## Data availability statement

The original contributions presented in the study are included in the article/supplementary material, further inquiries can be directed to the corresponding author.

## Ethics statement

Written informed consent was obtained from the minor(s)' legal guardian/next of kin for the publication of any potentially identifiable images or data included in this article.

## Author contributions

WH: Writing – original draft, Writing – review & editing. XX: Data curation, Writing – review & editing. YZ: Supervision, Writing – original draft. YP: Data curation, Formal analysis, Writing – original draft. LS: Data curation, Writing – original draft. LL: Data curation, Writing – review & editing. JG: Supervision, Writing – review & editing. LK: Funding acquisition, Writing – review & editing.
